# Crystal structure, Hirshfeld analysis and electrochemical properties of poly[di­aqua­bis­[μ_6_-2-cyano-2-(oxido­imino)­acetato]­copper(II)disodium]

**DOI:** 10.1107/S2056989025008126

**Published:** 2025-09-19

**Authors:** Maksym O. Plutenko, Irina A. Golenya, Vladyslav M. Plavkov, Tetiana Matus, József S. Pap, Sergiu Shova

**Affiliations:** a Department of Chemistry National Taras Shevchenko University Volodymyrska Street 64 01601 Kyiv Ukraine; b Innovation Development Center ABN LLC, Pirogov Str. 2/37, Kyiv, 01030, Ukraine; c HUN-REN Centre for Energy Research, Institute for Energy Security and, Environmental Safety, Surface Chemistry and Catalysis Department, H-1121, Budapest, Konkoly-Thege Rd. 29-33, Hungary; d Department of Inorganic Polymers "Petru Poni" Institute of Macromolecular Chemistry Romanian Academy of Science Aleea Grigore Ghica Voda 41-A Iasi 700487 Romania; University of Neuchâtel, Switzerland

**Keywords:** crystal structure, copper(II) complex, oxime-based ligand, Hirshfeld surface analysis, electrochemical properties

## Abstract

The title compound, [Na_2_Cu(C_3_N_2_O_3_)_2_(H_2_O)_2_]_*n*_, is a three-dimensional coordination polymer composed of divalent copper-centered complex anions, linked by sodium and water mol­ecules. The copper(II) ion adopts a distorted octahedral coordination geometry, defined by two nitro­gen atoms from oxime groups and two oxygen atoms from carboxyl­ate groups, originating from two *trans*-oriented, doubly deprotonated residues of 2-cyano-2-(hy­droxy­imino)­acetic acid. The axial Cu—O bonds are much longer than the equatorial Cu—O and Cu—N bonds due to the Jahn–Teller effect. Each complex anionic unit is linked to four neighboring anions *via* Cu—O bonds, forming a two-dimensional anionic network parallel to the *bc* plane.

## Abstract 2

The synthesis and mol­ecular crystal structure of an anionic copper(II) complex with 2-cyano-2-(hy­droxy­imino)­propionic acid are reported. The complex exhibits distorted octahedral coordination geometry around the copper(II) center, featuring two bidentate dianionic ligands arranged in a *trans*-configuration. Coordination occurs *via* the nitro­gen atom of the oxime group and the oxygen atom of the carboxyl­ate group, with the latter binding in a monodentate manner.

## 1. Chemical context

Mononuclear 3*d*-metal complexes often contain non-coordinated donor atoms or chelating functionalities, which can be further employed as binding sites for exo-coordination in the design of homo- and heteropolynuclear architectures. Such systems are employed extensively in bio-inorganic modeling, mol­ecular magnetism, and multi-electron photo- and electrocatalytic processes (Fritsky *et al.*, 2001 ▸, 2003 ▸; Wörl *et al.*, 2005*a* ▸). In particular, multidentate ligands containing mixed donor sets, specifically combinations of oxime groups with either carboxyl­ate or amide functionalities, have attracted considerable attention due to their rich and versatile coordination chemistry. Firstly, the inherent acidic nature of these groups facilitates the formation of negatively charged ligabds or acidoligands, which can readily yield anionic complexes of varying charge states (Fritsky *et al.*, 2004 ▸; Kanderal *et al.*, 2005 ▸). Secondly, these functional groups are characterized by diverse coordination modes, ranging from monodentate to bridging inter­actions (Strotmeyer *et al.*, 2003 ▸; Wörl *et al.*, 2005*b* ▸). Such versatility significantly broadens the spectrum of attainable complex structures and strongly favors the assembly of polynuclear systems exhibiting various types of magnetic inter­actions.

A particularly noteworthy feature of these donor groups is their ability, in negatively charged forms, to effectively stabilize higher oxidation states of 3*d* transition metals, driven by their pronounced σ-donor character. Indeed, our earlier studies have demonstrated that ligands featuring a donor set of 2×{N(oxime), N(amide)} effectively stabilize high-valent nickel(III) and copper(III) species (Kanderal *et al.*, 2005 ▸; Fritsky *et al.*, 2006 ▸). These properties underpin considerable inter­est in such ligand systems and their complexes, particularly as potential catalysts for electrochemical and photochemical water oxidation (Kondo *et al.*, 2021 ▸; Beil *et al.*, 2022 ▸). In addition, coordination polymers and metal–organic frameworks (MOFs) featuring such anionic complexes are of particular inter­est as promising components of hybrid electrode materials. In light of the growing global emphasis on sustainable catalytic technologies, recent decades have seen a marked increase in reports on mol­ecular coordination compounds and MOFs exhibiting relevant catalytic functionality (Singh *et al.*, 2021 ▸; Liu *et al.*, 2023 ▸).

Although carboxyl­ate groups generally show a somewhat lower tendency to stabilize high-valent oxidation states compared to amide groups, ligands featuring {N(oxime), O(carboxyl­ate)} donor sets also exhibit significant coordination potential. Notably, coordination compounds based on 2-cyano-2-(hy­droxy­imino)­acetic acid (H_2_aaco) have been reported, demonstrating their ability to form anionic complexes with metals such as copper(II), nickel(II), and palladium(II) (Eddings *et al.*, 2004 ▸; Golenya *et al.*, 2012 ▸; Opalade, *et al.*, 2019 ▸). In particular, the deprotonated form of H_2_aaco has proven to be an effective chelating ligand for Cu^II^ and Ni^II^ ions, forming a variety of mono- and polynuclear complexes depending on the stoichiometry and reaction conditions (Sliva *et al.*, 1998 ▸; Mokhir *et al.*, 2002 ▸). Inter­estingly, some of these complexes were obtained via hydrolysis of the corresponding ethyl ester precursors (Eddings *et al.*, 2004 ▸).

This work is part of a broader study aimed at synthesizing polynuclear assemblies, coordination polymers, and MOFs based on mononuclear copper(II) complexes derived from this ligand; investigating their mol­ecular and crystal structures along with their supra­molecular organization; and evaluating their electrochemical behavior and potential electrocatalytic activity in water oxidation (Sliva *et al.*, 1998 ▸; Golenya *et al.*, 2012 ▸).
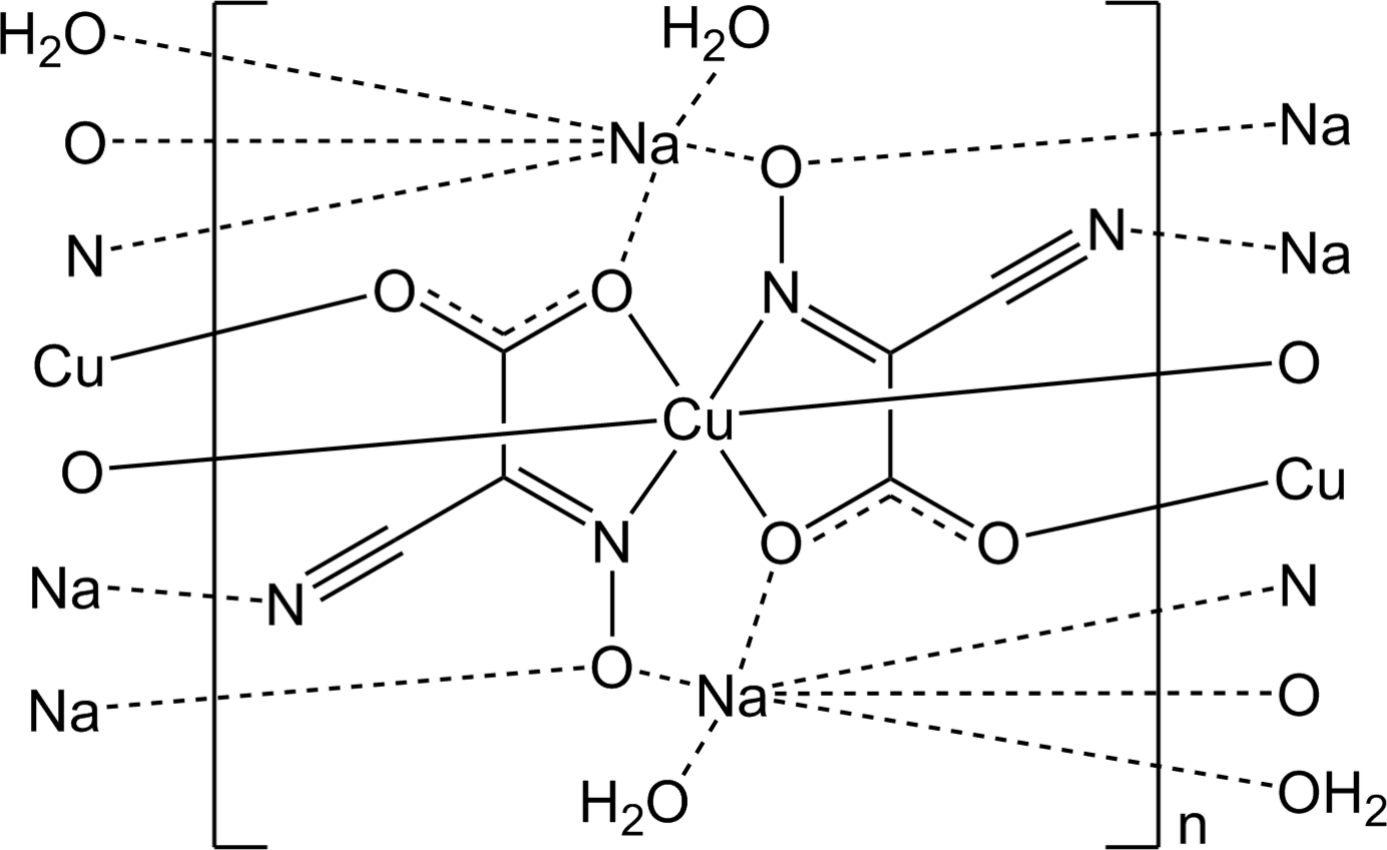


## 2. Structural commentary

**Figure 1:**
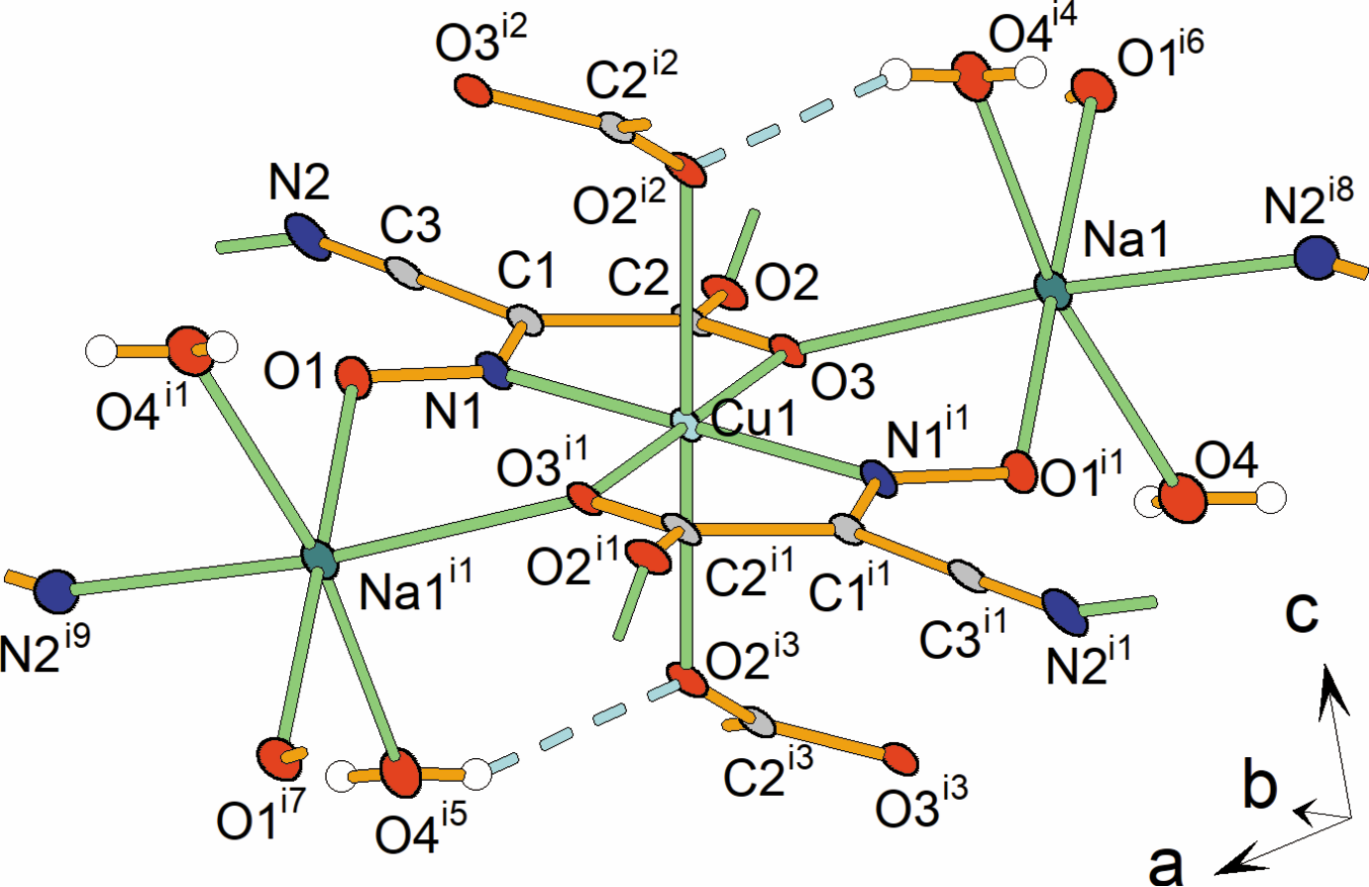
The [Na_2_Cu(C_3_N_2_O_3_)_2_(H_2_O)_2_] complex monomer with displacement ellipsoids shown at the 50% probability level. Symmetry codes: (i1) −*x* + 1, −*y* + 1, −*z* + 1; (i2) −*x* + 1, *y* + 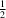, −*z* + 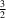; (i3) *x*, −*y* + 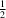, *z* − 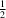; (i4) *x*, −*y* + 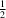, *z* + 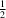; (i5) −*x* + 1, *y* + 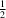, −*z* + 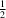; (i6) −*x* + 1, *y* − 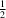, −*z* + 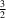; (i7) *x*, −*y* + 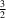, *z* − 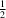; (i8) *x* − 1, −*y* + 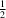, *z* − 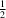; (i9) −*x* + 2, *y* + 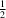, −*z* + 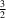.

The title compound, [Na_2_Cu(C_3_N_2_O_3_)_2_(H_2_O)_2_]_*n*_, is a three-dimensional coordination polymer composed of Cu^II^-centered complex anions, sodium cations and water mol­ecules (Fig. 1 ▸). The copper(II) ion adopts a distorted octahedral coordination geometry, defined by two nitro­gen atoms from oxime groups and two oxygen atoms from carboxyl­ate groups, originating from two *trans*-oriented, doubly deprotonated residues of 2-cyano-2-(hy­droxy­imino)­acetic acid. The axial positions are occupied by carboxyl­ate oxygen atoms (O2) from adjacent ligands, which are not involved in chelation via the oxime nitro­gen atom.

The Cu—N and Cu—O bond lengths are consistent with those typically observed in distorted octahedral Cu^II^ complexes containing deprotonated oxime and carboxyl­ate donors (Sliva *et al.*, 1998 ▸; Kanderal *et al.*, 2005 ▸). The axial Cu—O2 bonds [2.409 (4) Å] are much longer than equatorial Cu—O3 [1.974 (4) Å] and Cu—N1 [1.972 (5) Å] due to the Jahn–Teller effect. The bite angles around the Cu center deviate slightly from an ideal square-planar arrangement [*e.g*., O2—Cu1—N1 = 88.87 (16)°], reflecting the constraints imposed by the formation of five-membered chelate rings. Bond distances within the coordinated 2-oximino­carboxyl­ate ligands (C—O, N—O, and C—N) fall within the expected ranges for copper(II) complexes with cyanoxime and carboxyl­ate ligands (Onindo *et al.*, 1995 ▸; Duda *et al.*, 1997 ▸; Fritsky *et al.*, 2004 ▸).

## 3. Supra­molecular features

**Figure 2:**
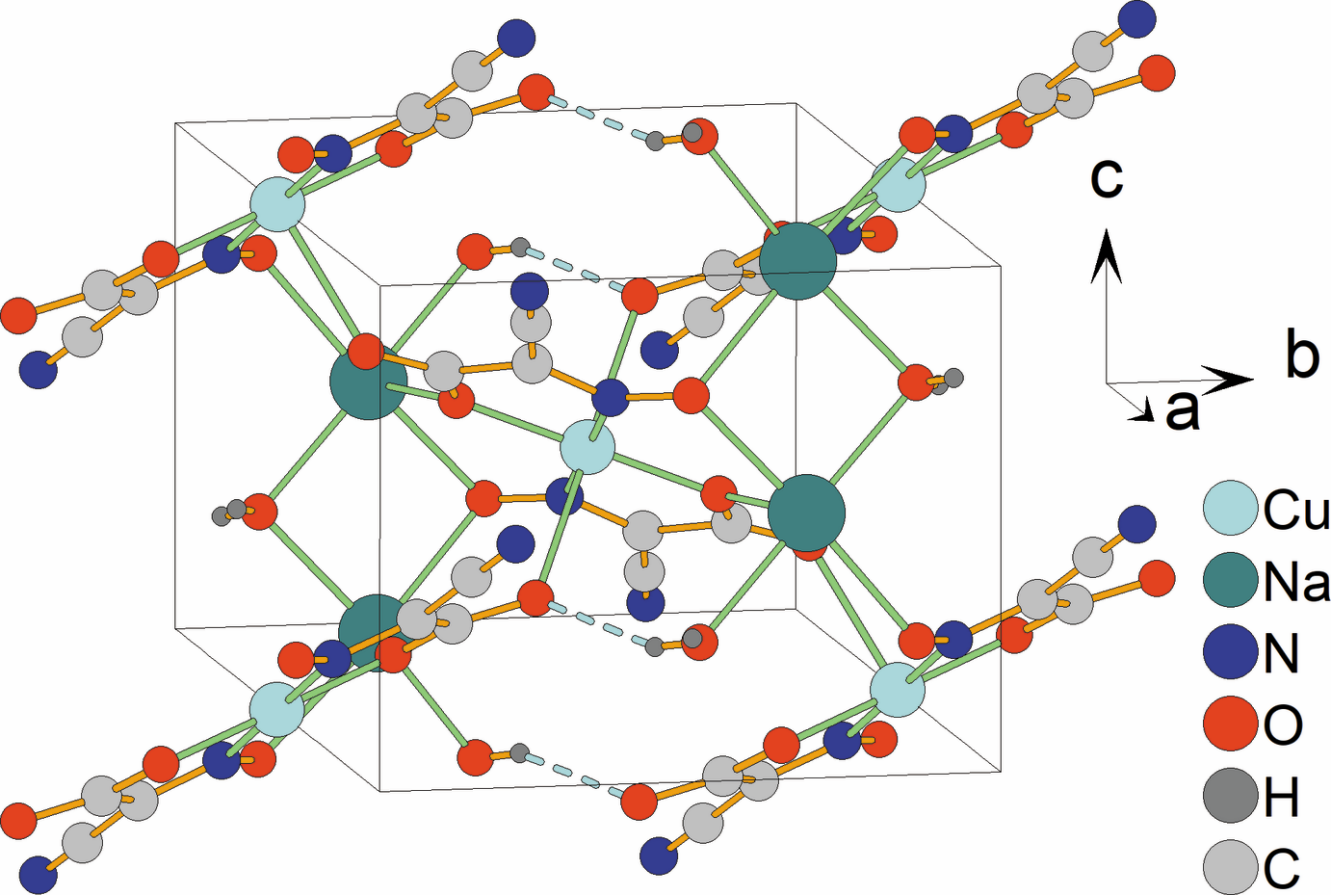
Crystal packing of the title compound.

In the crystal, the [Cu(C_3_N_2_O_3_)_2_]^2−^ complex anions are connected to each other through bridging coordinated carb­oxy­lic groups *via* Cu1—O2 bonds. As a result, each [Cu(C_3_N_2_O_3_)_2_]^2−^ unit is linked to four neighboring units, forming a two-dimensional anionic network parallel to the *bc* plane (Fig. 2 ▸). These individual layers are further connected through Na cations *via* O1—Na1, O3—Na1, and N2—Na1 ionic bonds. The carb­oxy­lic group of the ligand thus exhibits a μ_3_-coordination mode, binding to two copper ions and one sodium cation. In addition, the carboxylic group is connected to the water molecule *via* an O4—H4*A*⋯O2 hydrogen bond (Table 1 ▸) in which the water oxygen atom acts as hydrogen-bond donor and the oxygen atom of the carboxylic group acts as acceptor.

The sodium cation adopts a distorted octa­hedral environment, defined by two oxygen atoms from oxime groups, one oxygen atom from a carb­oxy­lic group, one nitro­gen atom from a cyano group, and two oxygen atoms from neighboring water mol­ecules. Individual sodium cations are inter­connected *via* bridging oxime groups and water mol­ecules through Na1—O1 and Na1—O4 inter­actions, forming supra­molecular chains along the *c*-axis direction.

## 4. Hirshfeld analysis

**Figure 3:**
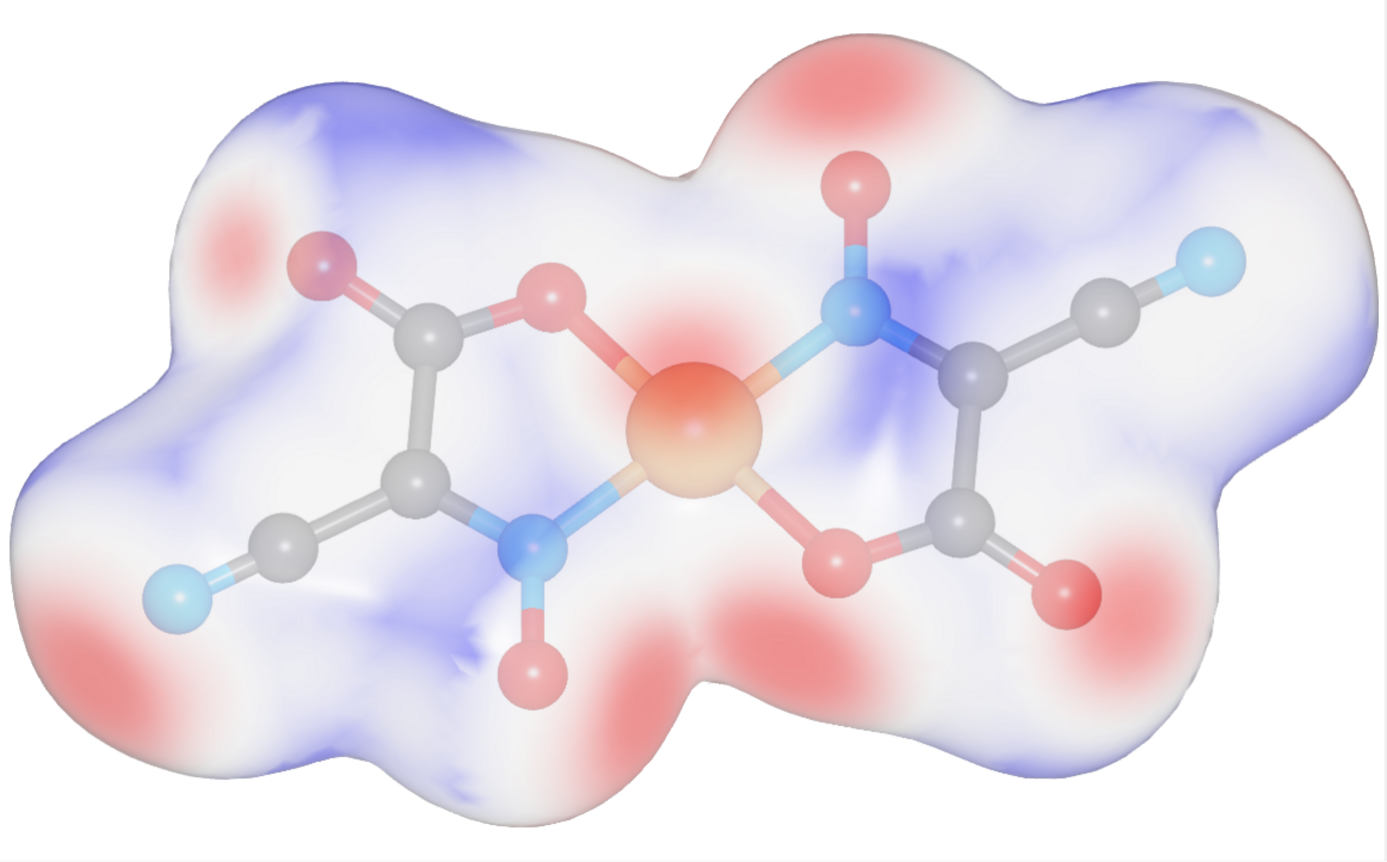
The Hirshfeld surfaces of the [Cu(C_3_N_2_O_3_)_2_]^2−^ complex anion.

The Hirshfeld surface analysis (Spackman & Jayatilaka, 2009 ▸) and the associated two-dimensional fingerprint plots (McKinnon *et al.*, 2007 ▸) were performed with *CrystalExplorer 25* (Spackman *et al.*, 2021 ▸). The Hirshfeld surfaces of the [Cu(C_3_N_2_O_3_)_2_]^2−^ complex anion are color-mapped with the normalized contact distance (*d*_norm_) from red (distances shorter than the sum of the van der Waals radii) through white to blue (distances longer than the sum of the van der Waals radii). According to the Hirshfeld surface (Fig. 3 ▸), the most noticeable inter­molecular inter­actions are O1⋯Na1, O2⋯Cu1, O3⋯Na1 and N2⋯Na1 contacts.

**Figure 4:**
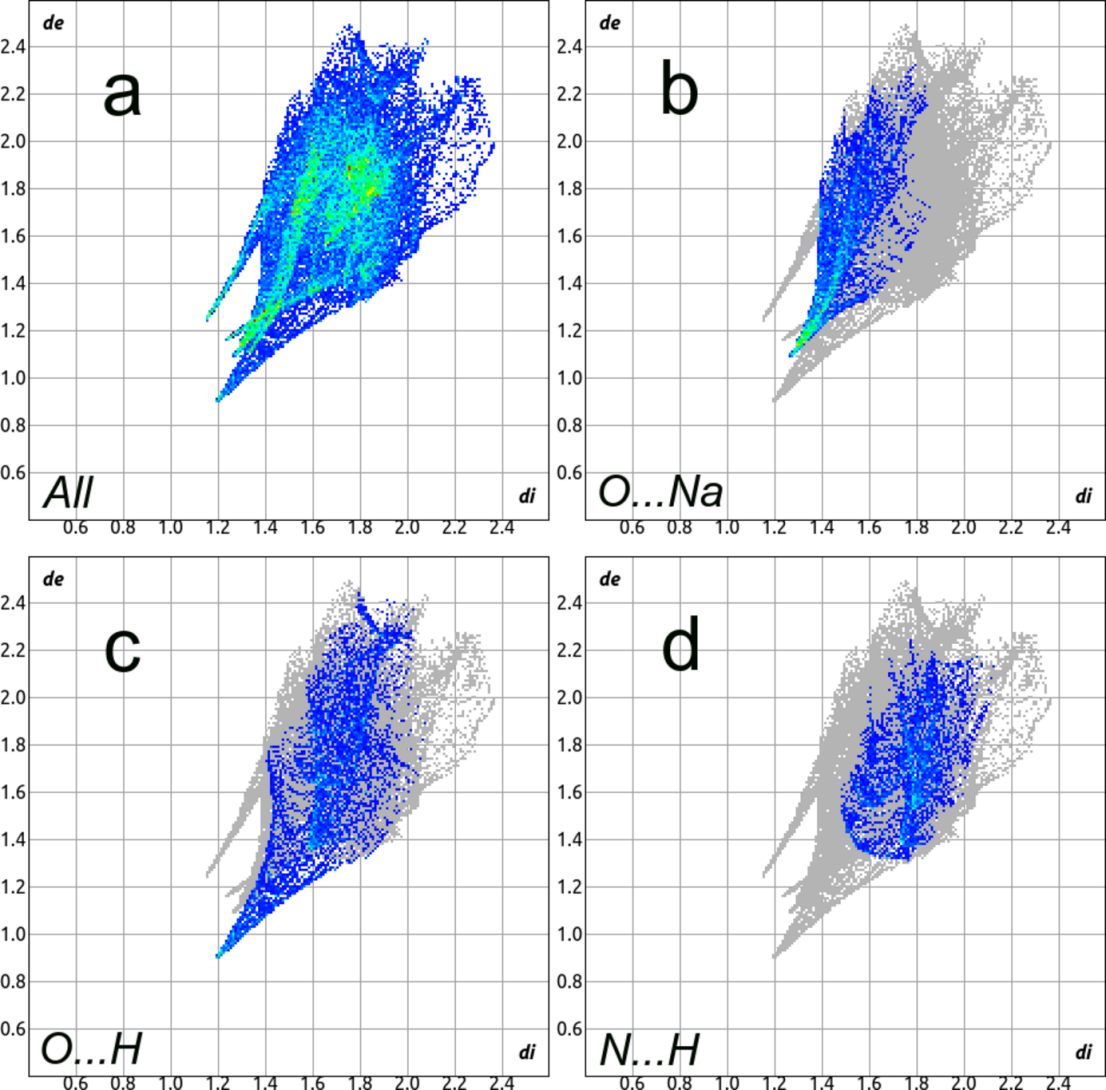
(*a*) Full two-dimensional fingerprint plot of the [Cu(C_3_N_2_O_3_)_2_]^2−^ complex anion delineated into (*b*) O⋯Na (14.8%), (*c*) O⋯H (12.8%) and (*d*) N⋯H (19.3%) contacts.

A fingerprint plot delineated into specific inter­atomic contacts contains information related to specific inter­molecular inter­actions. The blue color refers to the frequency of occurrence of the (*d*_i_, *d*_e_) pair with the full fingerprint plot outlined in gray. Fig. 4 ▸ shows the two-dimensional fingerprint plot of the sum of the contacts contributing to the Hirshfeld surface represented in normal mode. The most significant contribution to the Hirshfeld surface is from O⋯Na (14.8%) and O⋯H (12.8%) contacts. In addition, N⋯H (9.3%) is a significant contribution to the total Hirshfeld surface.

## 5. Redox Properties

**Figure 5:**
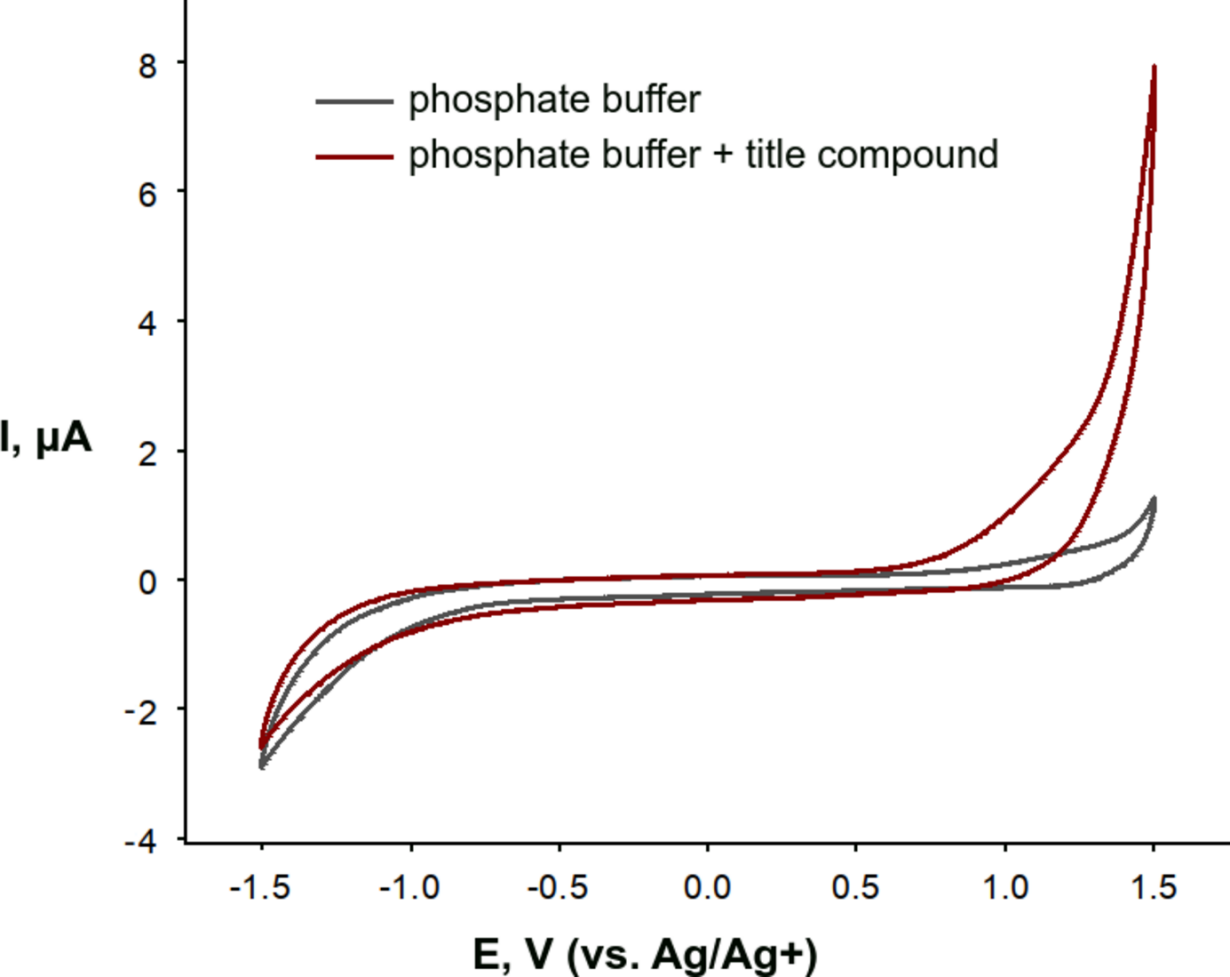
Cyclic voltammogram of the 0.1 *M* phosphate buffer (gray line) and the 0.1 m*M* title compound in the 0.1 *M* phosphate buffer (red line). Boron-doped diamond electrode, Ag/AgCl reference electrode (E(Ag/Ag^+^) = 0.210 V *versus* NHE).

Electrochemical investigations revealed that the complex exhibits no redox activity within the potential range of −1.5 V to +1.5 V (Fig. 5 ▸), effectively ruling out its utility as a photo- or electrocatalyst for water oxidation. This redox-inert behavior and the absence of any indication of accessible copper(III) states under these conditions is likely attributed to the strong π-acceptor effect of the nitrile groups. These effects offset the anti­cipated lowering of the Cu^3+/2+^ redox potential by the anionic nature and strong σ-donor capacity of the coordinating nitro­gen and oxygen atoms of the oxime and carboxyl­ate functionalities, respectively, thereby disfavoring the stabilization of the trivalent copper state.

## 6. Database survey

A search in the Cambridge Structural Database (CSD version 5.45, update of June 2024; Groom *et al.*, 2016 ▸) resulted in 14 hits dealing with metal complexes including a 2-cyano-2-(oxido­imino)­acetate fragment. There are four Cu (CSD refcodes CEFHOM, CEFHOM01, KIXHUX and SOQZIH), four Sb (SURXUB, SURXUB01, SURYAI and SURYAI01), three Ni (KIXJAF, KIXJOT and NEVKIJ), two Ag (YIYPOM and YIYPOM01) and one Pd (SABQES) complex structures.

Two of the four Cu complexes (CEFHOM and CEFHOM01) exhibit a distorted square-pyramidal CuO_3_N_2_ coordination geometry. One complex (SOQZIH) adopts a distorted octahedral CuO_5_N geometry. Another complex (KIXHUX) displays both distorted square-pyramidal and distorted octahedral coordination geometries, with CuO_3_N_2_ and CuO_6_ arrangements, respectively. The geometrical parameters of these complexes are similar to that of the title compound. As in the title compound, the axial Cu—O bonds (2.214–2.495 Å) are longer than the equatorial Cu—O (1.921–1.978 Å) and Cu—N (1.976–2.008 Å) bonds, consistent with the Jahn–Teller effect.

## 7. Synthesis and crystallization

The title compound was obtained serendipitously during an attempt to synthesize a binuclear copper(II) complex with 2-cyano-2-(oxyimino)­acetate anions and 1,4-di­aza­bicyclo­octane as a bridging ligand (Petrusenko *et al.*, 1997 ▸). A solution of copper(II) chloride dihydrate (0.0340 g, 0.2 mmol) in methanol (5 mL) was added to a solution of ethyl 2-cyano-2-(oxyimino)­acetate (0.0568 g, 0.4 mmol) in methanol (5 mL). Separately, 1,4-di­aza­bicyclo­octane (0.0112 g, 0.1 mmol) was dissolved in methanol (3 mL) and added to the reaction mixture. Then, NaOH solution in methanol (4 ml, 0.1 *M*, 0.4 mmol) was added. The resulting mixture was stirred and heated (323 K) for 60 minutes, then cooled to room temperature, filtered, and left to stand for crystallization. X-ray quality crystals formed after one week; they were collected by filtration, washed with acetone, and air-dried. Yield: 0.0538 g (73%).

## 8. Electrochemistry

Cyclic voltammetry (CV) experiments were conducted in aqueous solutions containing 0.1 *M* phosphate buffer at pH 7.2 as the supporting electrolyte, using a BioLogic SP-150 galvanostat/potentiostat. A three-electrode setup was employed, comprising a boron-doped diamond electrode (3 mm diameter, polished first with a diamond paste, then rinsed it with MilliQ water, next polished with an Al_2_O_3_ paste before rinsing again and finally, ultrasonicated the electrode in MilliQ water to remove any remaining particles prior to use), a platinum wire counter electrode, and an Ag/AgCl reference electrode. The electrolyte was purged with argon gas before each measurement and maintained under an inert argon atmosphere throughout the experiments. All potentials are reported *versus* the Ag/Ag^+^ couple, *E*(Ag/Ag^+^) = 0.210 V *vs* NHE.

## 9. Refinement

Crystal data, data collection and structure refinement details are summarized in Table 2 ▸. The non-hydrogen atoms were refined anisotropically. The hydrogen atoms were located in a difference-Fourier map, and their positions and isotropic thermal parameters were freely refined.

## Supplementary Material

Crystal structure: contains datablock(s) I. DOI: 10.1107/S2056989025008126/tx2103sup1.cif

Structure factors: contains datablock(s) I. DOI: 10.1107/S2056989025008126/tx2103Isup2.hkl

Supporting information file. DOI: 10.1107/S2056989025008126/tx2103Isup3.cdx

Supporting information file. DOI: 10.1107/S2056989025008126/tx2103Isup4.mol

Supporting information file. DOI: 10.1107/S2056989025008126/tx2103Isup5.cdx

CCDC reference: 2487839

Additional supporting information:  crystallographic information; 3D view; checkCIF report

## Figures and Tables

**Table 1:** Hydrogen-bond geometry (Å, °)

*D*—H⋯*A*	*D*—H	H⋯*A*	*D*⋯*A*	*D*—H⋯*A*
O4—H4*A*⋯O2^i^	0.70 (9)	2.10 (9)	2.753 (6)	155 (9)

Symmetry code: (i) 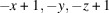.

**Table 2:** Experimental details

Crystal data	
Chemical formula	[Na_2_Cu(C_3_N_2_O_3_)_2_(H_2_O)_2_]
*M*_r_	369.65
Crystal system, space group	Monoclinic, *P*2_1_/*c*
Temperature (K)	100
*a*, *b*, *c* (Å)	9.1889 (4), 9.1459 (4), 7.1062 (3)
β (°)	99.090 (4)
*V* (Å^3^)	589.71 (4)
*Z*	2
Radiation type	Cu *K*α
μ (mm^−1^)	3.87
Crystal size (mm)	0.15 × 0.10 × 0.02

Data collection	
Diffractometer	XtaLAB Synergy, Dualflex, HyPix
Absorption correction	Multi-scan (*CrysAlis PRO*; Rigaku OD, 2024 ▸)
*T*_min_, *T*_max_	0.741, 1.000
No. of measured, independent and observed [*I* > 2σ(*I*)] reflections	1044, 1044, 982
*R*_int_	0.67
(sin θ/λ)_max_ (Å^−1^)	0.595

Refinement	
*R*[*F*^2^ > 2σ(*F*^2^)], *wR*(*F*^2^), *S*	0.052, 0.177, 1.22
No. of reflections	1044
No. of parameters	106
H-atom treatment	All H-atom parameters refined
Δρ_max_, Δρ_min_ (e Å^−3^)	1.70, −1.55

Computer programs: *CrysAlis PRO* (Rigaku OD, 2024 ▸), *SHELXT2018/2* (Sheldrick, 2015*a* ▸), *SHELXL2018/3* (Sheldrick, 2015*b* ▸), *DIAMOND* (Brandenburg, 2005 ▸) and *OLEX2* (Dolomanov *et al.*, 2009 ▸).

## supplementary crystallographic information

### Poly[diaquabis[µ6-2-cyano-2-(oxidoimino)acetato]copper(II)disodium] Crystal data

**Table:**

[Na_2_Cu(C_3_N_2_O_3_)_2_(H_2_O)_2_]	*F*(000) = 366
*M**_r_* = 369.65	*D*_x_ = 2.082 Mg m^−^^3^
Monoclinic, *P*2_1_/*c*	Cu *K*α radiation, λ = 1.54184 Å
*a* = 9.1889 (4) Å	Cell parameters from 2721 reflections
*b* = 9.1459 (4) Å	θ = 4.9–75.2°
*c* = 7.1062 (3) Å	µ = 3.87 mm^−^^1^
β = 99.090 (4)°	*T* = 100 K
*V* = 589.71 (4) Å^3^	Plate, clear light brown
*Z* = 2	0.15 × 0.10 × 0.02 mm

### Poly[diaquabis[µ6-2-cyano-2-(oxidoimino)acetato]copper(II)disodium] Data collection

**Table:**

XtaLAB Synergy, Dualflex, HyPix diffractometer	1044 independent reflections
Radiation source: micro-focus sealed X-ray tube, PhotonJet (Cu) X-ray Source	982 reflections with *I* > 2σ(*I*)
Detector resolution: 10.0000 pixels mm^-1^	*R*_int_ = 0.67
ω scans	θ_max_ = 66.6°, θ_min_ = 4.9°
Absorption correction: multi-scan (CrysAlisPro; Rigaku OD, 2024)	*h* = −10→10
*T*_min_ = 0.741, *T*_max_ = 1.000	*k* = −10→10
1044 measured reflections	*l* = −5→8

### Poly[diaquabis[µ6-2-cyano-2-(oxidoimino)acetato]copper(II)disodium] Refinement

**Table:**

Refinement on *F*^2^	0 restraints
Least-squares matrix: full	Hydrogen site location: difference Fourier map
*R*[*F*^2^ > 2σ(*F*^2^)] = 0.052	All H-atom parameters refined
*wR*(*F*^2^) = 0.177	*w* = 1/[σ^2^(*F*_o_^2^) + (0.0875*P*)^2^ + 2.7951*P*] where *P* = (*F*_o_^2^ + 2*F*_c_^2^)/3
*S* = 1.22	(Δ/σ)_max_ < 0.001
1044 reflections	Δρ_max_ = 1.70 e Å^−^^3^
106 parameters	Δρ_min_ = −1.55 e Å^−^^3^

### Poly[diaquabis[µ6-2-cyano-2-(oxidoimino)acetato]copper(II)disodium] Special details

**Table:**

Geometry. All esds (except the esd in the dihedral angle between two l.s. planes) are estimated using the full covariance matrix. The cell esds are taken into account individually in the estimation of esds in distances, angles and torsion angles; correlations between esds in cell parameters are only used when they are defined by crystal symmetry. An approximate (isotropic) treatment of cell esds is used for estimating esds involving l.s. planes.
Refinement. Refined as a 2-component twin.

### Poly[diaquabis[µ6-2-cyano-2-(oxidoimino)acetato]copper(II)disodium] Fractional atomic coordinates and isotropic or equivalent isotropic displacement parameters (Å^2^)

**Table:**

	*x*	*y*	*z*	*U*_iso_*/*U*_eq_	
Cu1	0.500000	0.500000	0.500000	0.0075 (4)	
Na1	0.2083 (2)	0.2433 (2)	0.5468 (3)	0.0106 (6)	
N1	0.6935 (5)	0.4743 (5)	0.6615 (7)	0.0096 (10)	
N2	0.9401 (5)	0.2608 (6)	0.9596 (7)	0.0160 (11)	
O1	0.7992 (4)	0.5681 (4)	0.6968 (6)	0.0115 (9)	
O2	0.5888 (4)	0.1131 (4)	0.7338 (5)	0.0110 (9)	
O3	0.4627 (4)	0.3009 (4)	0.5884 (5)	0.0087 (8)	
O4	0.1809 (5)	0.0760 (5)	0.2858 (6)	0.0149 (9)	
H4A	0.235 (9)	0.022 (9)	0.310 (12)	0.02 (2)*	
H4B	0.107 (11)	0.039 (12)	0.253 (16)	0.05 (3)*	
C1	0.7049 (6)	0.3431 (6)	0.7386 (8)	0.0087 (11)	
C2	0.5766 (6)	0.2432 (6)	0.6845 (8)	0.0093 (11)	
C3	0.8351 (6)	0.2974 (6)	0.8622 (8)	0.0106 (11)	

### Poly[diaquabis[µ6-2-cyano-2-(oxidoimino)acetato]copper(II)disodium] Atomic displacement parameters (Å^2^)

**Table:**

	*U*^11^	*U*^22^	*U*^33^	*U*^12^	*U*^13^	*U*^23^
Cu1	0.0072 (6)	0.0057 (6)	0.0103 (7)	−0.0002 (4)	0.0035 (5)	0.0016 (4)
Na1	0.0094 (10)	0.0102 (11)	0.0132 (11)	−0.0009 (8)	0.0043 (8)	0.0014 (9)
N1	0.009 (2)	0.007 (2)	0.014 (2)	−0.0010 (17)	0.0070 (19)	−0.0015 (19)
N2	0.013 (2)	0.018 (2)	0.019 (2)	−0.002 (2)	0.007 (2)	0.011 (2)
O1	0.0114 (19)	0.0081 (19)	0.016 (2)	−0.0035 (14)	0.0048 (16)	−0.0027 (15)
O2	0.0131 (19)	0.0083 (19)	0.0137 (19)	0.0021 (14)	0.0088 (15)	0.0000 (15)
O3	0.0089 (18)	0.0076 (17)	0.0109 (18)	−0.0004 (14)	0.0058 (15)	0.0007 (14)
O4	0.014 (2)	0.010 (2)	0.020 (2)	0.0036 (19)	0.0051 (18)	0.0002 (17)
C1	0.007 (2)	0.011 (3)	0.010 (2)	0.002 (2)	0.005 (2)	−0.001 (2)
C2	0.009 (2)	0.009 (3)	0.011 (2)	0.001 (2)	0.007 (2)	0.002 (2)
C3	0.012 (3)	0.010 (3)	0.012 (3)	−0.003 (2)	0.008 (2)	0.002 (2)

### Poly[diaquabis[µ6-2-cyano-2-(oxidoimino)acetato]copper(II)disodium] Geometric parameters (Å, º)

**Table:**

Cu1—N1^i^	1.972 (5)	Na1—N2^vi^	2.445 (5)
Cu1—N1	1.972 (5)	Na1—Na1^ii^	3.5552 (2)
Cu1—O3	1.974 (4)	Na1—Na1^iv^	3.5553 (2)
Cu1—O3^i^	1.974 (4)	Na1—H4A	2.67 (8)
Cu1—O2^ii^	2.409 (4)	N1—O1	1.290 (6)
Cu1—O2^iii^	2.409 (4)	N1—C1	1.316 (7)
Cu1—Na1	3.618 (2)	N2—C3	1.145 (8)
Cu1—Na1^i^	3.618 (2)	O2—C2	1.240 (7)
Na1—O3	2.368 (4)	O3—C2	1.271 (7)
Na1—O4	2.387 (5)	O4—H4A	0.70 (9)
Na1—O4^iv^	2.412 (5)	O4—H4B	0.76 (10)
Na1—O1^v^	2.435 (4)	C1—C3	1.430 (8)
Na1—O1^i^	2.437 (4)	C1—C2	1.493 (7)
			
N1^i^—Cu1—N1	180.0	O3—Na1—Na1^iv^	91.35 (10)
N1^i^—Cu1—O3	96.25 (17)	O4—Na1—Na1^iv^	141.45 (15)
N1—Cu1—O3	83.75 (17)	O4^iv^—Na1—Na1^iv^	41.92 (11)
N1^i^—Cu1—O3^i^	83.75 (17)	O1^v^—Na1—Na1^iv^	43.16 (10)
N1—Cu1—O3^i^	96.25 (17)	O1^i^—Na1—Na1^iv^	132.92 (14)
O3—Cu1—O3^i^	180.0	N2^vi^—Na1—Na1^iv^	95.44 (13)
N1^i^—Cu1—O2^ii^	88.87 (16)	Na1^ii^—Na1—Na1^iv^	176.04 (13)
N1—Cu1—O2^ii^	91.13 (16)	O3—Na1—Cu1	30.26 (9)
O3—Cu1—O2^ii^	87.20 (13)	O4—Na1—Cu1	109.47 (12)
O3^i^—Cu1—O2^ii^	92.80 (14)	O4^iv^—Na1—Cu1	77.37 (13)
N1^i^—Cu1—O2^iii^	91.13 (16)	O1^v^—Na1—Cu1	127.21 (11)
N1—Cu1—O2^iii^	88.87 (16)	O1^i^—Na1—Cu1	54.10 (9)
O3—Cu1—O2^iii^	92.80 (14)	N2^vi^—Na1—Cu1	135.92 (15)
O3^i^—Cu1—O2^iii^	87.20 (14)	Na1^ii^—Na1—Cu1	76.62 (5)
O2^ii^—Cu1—O2^iii^	180.0	Na1^iv^—Na1—Cu1	100.75 (6)
N1^i^—Cu1—Na1	61.17 (14)	O3—Na1—H4A	93.4 (18)
N1—Cu1—Na1	118.83 (14)	O4—Na1—H4A	14.7 (18)
O3—Cu1—Na1	37.20 (11)	O4^iv^—Na1—H4A	173.9 (17)
O3^i^—Cu1—Na1	142.80 (11)	O1^v^—Na1—H4A	89.3 (17)
O2^ii^—Cu1—Na1	98.13 (9)	O1^i^—Na1—H4A	94.9 (17)
O2^iii^—Cu1—Na1	81.87 (9)	N2^vi^—Na1—H4A	91.0 (18)
N1^i^—Cu1—Na1^i^	118.83 (14)	Na1^ii^—Na1—H4A	51.8 (17)
N1—Cu1—Na1^i^	61.17 (14)	Na1^iv^—Na1—H4A	132.1 (17)
O3—Cu1—Na1^i^	142.80 (11)	Cu1—Na1—H4A	106.9 (17)
O3^i^—Cu1—Na1^i^	37.20 (11)	O1—N1—C1	121.2 (5)
O2^ii^—Cu1—Na1^i^	81.87 (9)	O1—N1—Cu1	128.0 (4)
O2^iii^—Cu1—Na1^i^	98.13 (9)	C1—N1—Cu1	110.8 (4)
Na1—Cu1—Na1^i^	180.0	C3—N2—Na1^vii^	151.3 (4)
O3—Na1—O4	102.74 (16)	N1—O1—Na1^iii^	118.2 (3)
O3—Na1—O4^iv^	88.32 (16)	N1—O1—Na1^i^	113.4 (3)
O4—Na1—O4^iv^	167.6 (3)	Na1^iii^—O1—Na1^i^	93.72 (13)
O3—Na1—O1^v^	101.37 (14)	C2—O2—Cu1^v^	127.4 (3)
O4—Na1—O1^v^	98.50 (16)	C2—O3—Cu1	112.6 (3)
O4^iv^—Na1—O1^v^	84.57 (15)	C2—O3—Na1	133.4 (3)
O3—Na1—O1^i^	81.24 (14)	Cu1—O3—Na1	112.54 (17)
O4—Na1—O1^i^	85.07 (15)	Na1—O4—Na1^ii^	95.62 (15)
O4^iv^—Na1—O1^i^	91.19 (15)	Na1—O4—H4A	106 (7)
O1^v^—Na1—O1^i^	174.92 (17)	Na1^ii^—O4—H4A	120 (7)
O3—Na1—N2^vi^	166.03 (18)	Na1—O4—H4B	120 (8)
O4—Na1—N2^vi^	79.32 (18)	Na1^ii^—O4—H4B	106 (8)
O4^iv^—Na1—N2^vi^	88.61 (18)	H4A—O4—H4B	109 (10)
O1^v^—Na1—N2^vi^	91.89 (17)	N1—C1—C3	121.7 (5)
O1^i^—Na1—N2^vi^	85.20 (16)	N1—C1—C2	116.3 (5)
O3—Na1—Na1^ii^	87.77 (10)	C3—C1—C2	121.9 (5)
O4—Na1—Na1^ii^	42.46 (11)	O2—C2—O3	125.5 (5)
O4^iv^—Na1—Na1^ii^	134.16 (15)	O2—C2—C1	118.8 (5)
O1^v^—Na1—Na1^ii^	140.81 (14)	O3—C2—C1	115.7 (5)
O1^i^—Na1—Na1^ii^	43.12 (10)	N2—C3—C1	179.3 (6)
N2^vi^—Na1—Na1^ii^	84.62 (13)		
			
C1—N1—O1—Na1^iii^	−94.0 (5)	Cu1^v^—O2—C2—C1	99.9 (5)
Cu1—N1—O1—Na1^iii^	84.8 (4)	Cu1—O3—C2—O2	−169.5 (4)
C1—N1—O1—Na1^i^	157.8 (4)	Na1—O3—C2—O2	25.6 (8)
Cu1—N1—O1—Na1^i^	−23.4 (5)	Cu1—O3—C2—C1	9.9 (5)
O1—N1—C1—C3	−1.0 (8)	Na1—O3—C2—C1	−155.1 (3)
Cu1—N1—C1—C3	180.0 (4)	N1—C1—C2—O2	171.1 (5)
O1—N1—C1—C2	−178.9 (4)	C3—C1—C2—O2	−6.8 (8)
Cu1—N1—C1—C2	2.1 (6)	N1—C1—C2—O3	−8.3 (7)
Cu1^v^—O2—C2—O3	−80.7 (6)	C3—C1—C2—O3	173.8 (5)

Symmetry codes: (i) −*x*+1, −*y*+1, −*z*+1; (ii) *x*, −*y*+1/2, *z*−1/2; (iii) −*x*+1, *y*+1/2, −*z*+3/2; (iv) *x*, −*y*+1/2, *z*+1/2; (v) −*x*+1, *y*−1/2, −*z*+3/2; (vi) *x*−1, −*y*+1/2, *z*−1/2; (vii) *x*+1, −*y*+1/2, *z*+1/2.

### Poly[diaquabis[µ6-2-cyano-2-(oxidoimino)acetato]copper(II)disodium] Hydrogen-bond geometry (Å, º)

**Table:**

*D*—H···*A*	*D*—H	H···*A*	*D*···*A*	*D*—H···*A*
O4—H4*A*···O2^viii^	0.70 (9)	2.10 (9)	2.753 (6)	155 (9)

Symmetry code: (viii) −*x*+1, −*y*, −*z*+1.
